# Supplementary material for: MetaQTL: a package of new computational methods for the meta-analysis of QTL mapping experiments
Source: BMC Bioinformatics. 2007 Feb 8;8:49. doi: 10.1186/1471-2105-8-49 (PMC1808479; doi:10.1186/1471-2105-8-49)
Supplement: Additional File 7 — MetaQTL Package : jar file and tutorial. This Zip archive contains both the MetaQTL JAR file and the files of the tutorial. [file 1471-2105-8-49-S7.zip › org.inra.metaqtl/doc/org/metaqtl/util/OffspringManager.html]

OffspringManager


|  |  |  |
| --- | --- | --- |
| **Overview** \| **Package** \| **Class** \| **Use** \| **Tree** \| **Deprecated** \| **Index** \| **Help** | |  |
| **PREV CLASS**   **NEXT CLASS** | **FRAMES**    **NO FRAMES**     **All Classes** |  |
| SUMMARY: NESTED \| FIELD \| CONSTR \| METHOD | DETAIL: FIELD \| CONSTR \| METHOD |  |


---


## org.metaqtl.util Class OffspringManager

```
java.lang.Object
  org.metaqtl.util.OffspringManager
```

---

``` public final class OffspringManager extends java.lang.Object ```

Class Description Here

**Author:**
:   Jean-Baptiste Veyrieras

---

| **Nested Class Summary** | |
| --- | --- |
| `class` | `OffspringManager.HaldaneWaddingtonProportion` |


**Constructor Summary**

`OffspringManager()`


| **Method Summary** | |
| --- | --- |
| `static double` | `fisherVariance(double r, int size, int type, int gen)`             Returns the inverse of the Fisher Information depending on the type of cross for a given recombination rate and a given population size. |
| `static double` | `r2R(double r, int type, int gen)`             This routine returns the recombination rate r for a given value of the propertion of recombibant genotypes R according to the cross design. |
| `static double` | `R2r(double R, int type, int gen)`             This routine returns the recombination rate r for a given value of the propertion of recombibant genotypes R according to the cross design. |
| `static double` | `selfingIndividualInformation(OffspringManager.HaldaneWaddingtonProportion p)`             This routine returns the individual means amount of information for the given generation propertions for a self-fertilization design. |
| `static void` | `selfingRecurrence(OffspringManager.HaldaneWaddingtonProportion p, double r)`             Given the current proportion C,D,E,F and G and the recombination rate r this routine computes the next generation proportions following the recurrence formula of Haldane and Waddington, 1931, Inbreeding and linkage, Genetics 16:357-374 (see section self-fertilization p358). |

**Methods inherited from class java.lang.Object**

`clone, equals, finalize, getClass, hashCode, notify, notifyAll, toString, wait, wait, wait`

**Constructor Detail**

### OffspringManager

```
public OffspringManager()
```


**Method Detail**

### fisherVariance

```
public static double fisherVariance(double r,
                                    int size,
                                    int type,
                                    int gen)
```

:   Returns the inverse of the Fisher Information depending on
    the type of cross for a given recombination rate and a given
    population size.

    :   **Parameters:**: `r` - the recombination rate estimate.: `size` -: `type` - **Returns:**: the variance for the recombination rate estimate.

---


### selfingRecurrence

```
public static void selfingRecurrence(OffspringManager.HaldaneWaddingtonProportion p,
                                     double r)
```

:   Given the current proportion C,D,E,F and G and the recombination
    rate r this routine computes the next generation proportions following
    the recurrence formula of Haldane and Waddington,
    1931, Inbreeding and linkage, Genetics 16:357-374
    (see section self-fertilization p358).
    Cn+1 = Cn + 0.5\*En + 0.25\*(1-r)^2\*Fn + 0.25\*r^2\*Gn
    Dn+1 = p.D + 0.5\*En + 0.25\*r^2Fn + 0.25\*(1-r)^2Gn
    En+1 = 0.5\*En + 0.5\*r\*(1-r)\*(Fn + Gn)
    Fn+1 = 0.5\*(1-r)^2Fn + 0.5\*r^2Gn
    Gn+1 = 0.5\*r^2Fn + 0.5\*(1-r)^2\*Gn
    This also computes the next generation derivates dC,dD,dE,dF and dG which can
    be easily obtained from the above equations.

    :   **Parameters:**: `proportion` -

---


### selfingIndividualInformation

```
public static double selfingIndividualInformation(OffspringManager.HaldaneWaddingtonProportion p)
```

:   This routine returns the individual means amount of information
    for the given generation propertions for a self-fertilization
    design. This assumes that the 4 zygotic classes can be discriminated
    without uncertainty - i.e the case where markers are codominants.

    :   **Parameters:**: `p` -

---


### R2r

```
public static double R2r(double R,
                         int type,
                         int gen)
```

:   This routine returns the recombination rate r for a given
    value of the propertion of recombibant genotypes R according
    to the cross design.
    type :
    IBioCrossTypes.RILSe Recombinant Inbred Lines via selfing
    R = 2r/(1+2r) (Haldane and Waddington 1931)
    IBioCrossTypes.RILSi Recombinant Inbred Lines via sib-mating
    R = 4r/(1+6r) (Haldane and Waddington 1931)
    IBioCrossTypes.IRISe High Recombinant Inbred Lines via selfing
    R = 0.5(1-((1-2r)/(1+2r))(1-r)^t) (Winkler et al. 2003)
    IBioCrossTypes.IRISi High Recombinant Inbred Lines via sib-mating
    R = 0.5(1-((1-2r)/(1+6r))(1-r)^t) (Winkler et al. 2003)

    :   **Parameters:**: `R` - the proportion of recombinant genotypes.: `size` - the population size.: `type` - the type of pedigree.: `gen` - the number of random mating generations (only for IRISi and IRISe). **Returns:**: the recombination rate r.

---


### r2R

```
public static double r2R(double r,
                         int type,
                         int gen)
```

:   This routine returns the recombination rate r for a given
    value of the propertion of recombibant genotypes R according
    to the cross design.
    type :
    IBioCrossTypes.RILSe Recombinant Inbred Lines via selfing
    R = 2r/(1+2r) (Haldane and Waddington 1931)
    IBioCrossTypes.RILSi Recombinant Inbred Lines via sib-mating
    R = 4r/(1+6r) (Haldane and Waddington 1931)
    IBioCrossTypes.IRISe High Recombinant Inbred Lines via selfing
    R = 0.5(1-((1-2r)/(1+2r))(1-r)^t) (Winkler et al. 2003)
    IBioCrossTypes.IRISi High Recombinant Inbred Lines via sib-mating
    R = 0.5(1-((1-2r)/(1+6r))(1-r)^t) (Winkler et al. 2003)

    :   **Parameters:**: `R` - the proportion of recombinant genotypes.: `size` - the population size.: `type` - the type of pedigree.: `gen` - the number of random mating generations (only for IRISi and IRISe). **Returns:**: the recombination rate r.


---


|  |  |  |
| --- | --- | --- |
| **Overview** \| **Package** \| **Class** \| **Use** \| **Tree** \| **Deprecated** \| **Index** \| **Help** | |  |
| **PREV CLASS**   **NEXT CLASS** | **FRAMES**    **NO FRAMES**     **All Classes** |  |
| SUMMARY: NESTED \| FIELD \| CONSTR \| METHOD | DETAIL: FIELD \| CONSTR \| METHOD |  |


---
